# Role of seminal plasma in the anti-HIV-1 activity of candidate microbicides

**DOI:** 10.1186/1471-2334-6-150

**Published:** 2006-10-16

**Authors:** A Robert Neurath, Nathan Strick, Yun-Yao Li

**Affiliations:** 1 Biochemical Virology Laboratory, The Lindsley F. Kimball Research Institute of the New York Blood Center, New York, NY 10021, USA

## Abstract

**Background:**

Evaluation of microbicides for prevention of HIV-1 infection in macaque models for vaginal infection has indicated that the concentrations of active compounds needed for protection by far exceed levels sufficient for complete inhibition of infection *in vitro*. These experiments were done in the absence of seminal plasma (SP), a vehicle for sexual transmission of the virus. To gain insight into the possible effect of SP on the performance of selected microbicides, their anti-HIV-1 activity in the presence, and absence of SP, was determined.

**Methods:**

The inhibitory activity of compounds against the X4 virus, HIV-1 IIIB, and the R5 virus, HIV-1 BaL was determined using TZM-bl indicator cells and quantitated by measuring β-galactosidase induced by infection. The virucidal properties of cellulose acetate 1,2-benzene-dicarboxylate (CAP), the only microbicide provided in water insoluble, micronized form, in the presence of SP was measured.

**Results:**

The HIV-1 inhibitory activity of the polymeric microbicides, poly(naphthalene sulfonate), cellulose sulfate, carrageenan, CAP (in soluble form) and polystyrene sulfonate, respectively, was considerably (range ≈ 4 to ≈ 73-fold) diminished in the presence of SP (33.3%). Formulations of micronized CAP, providing an acidic buffering system even in the presence of an SP volume excess, effectively inactivated HIV-1 infectivity.

**Conclusion:**

The data presented here suggest that the *in vivo *efficacy of polymeric microbicides, acting as HIV-1 entry inhibitors, might become at least partly compromised by the inevitable presence of SP. These possible disadvantages could be overcome by combining the respective polymers with acidic pH buffering systems (built-in for formulations of micronized CAP) or with other anti-HIV-1 compounds, the activity of which is not affected by SP, e.g. reverse transcriptase and zinc finger inhibitors.

## Background

Sexual virus transmission plays the major role in the worldwide HIV-1 epidemic [1]. In the absence of effective anti-HIV-1 vaccines, great emphasis has been put on the development of topical microbicides to be applied vaginally in the form of gels, creams or solid dosage formulations expected to inactivate HIV-1 infectivity or to interfere with steps in the virus life cycle, preferably blocking virus entry into susceptible cells. The model of choice for evaluating candidate anti-HIV-1 microbicides *in vivo *are female rhesus macaques to whom anti-HIV-1 products and either simian immunodeficiency virus (SIV) or HIV-1/SIV hybrid viruses (SHIVs) are consecutively applied in the vagina [2-7]. Results obtained in this animal model have indicated that the concentrations of anti-HIV-1 compounds in microbicide formulations adequate to prevent vaginal infection exceed by several orders of magnitude concentrations sufficient for complete inhibition of infection in *in vitro *systems [8-10]. The macaque model overlooks the role of human seminal plasma (SP), a common source of male to female sexual transmission of HIV-1, in infection and the ultimate effectiveness of microbicides. Because of impediments for including SP into the macaque model studies, the effect of this "natural diluent for HIV-1" on virus inhibitory activity of several candidate microbicides was investigated. They included the polymers: carrageenan, poly(naphthalene sulfonate) (PRO 2000), cellulose sulfate, cellulose acetate 1,2-benzenedicarboxylate (CAP) and polystyrene sulfonate, some of which are being evaluated in phase III clinical trials for efficacy [10-12]. Antiretroviral drugs specifically targeted to HIV-1 reverse transcriptase, UC781 [12,13] and TMC 120 [14,15], respectively, and to the zinc fingers of the HIV-1 nucleocapsid protein NCp7 [16-18] were included in control experiments.

## Methods

### Reagents

Aquateric (the micronized form of CAP containing ≈ 66% CAP and ≈ 34% of Poloxamer and distilled acetylated monoglycerides) was obtained from the FMC Biopolymer Corporation, Philadelphia, PA. The following polymers were obtained from commercial sources different from proprietary products being developed as microbicides: carrageenans κ and λ (Sigma, St. Louis, MO; mixed at a 1:1 (w/w) ratio in all experiments); cellulose sulfate (Across Organics, Piscataway, NJ); poly(napthalene sulfonate) (BASF, Parsippany, NJ); and polystyrene-4-sulfonate (Polysciences, Inc., Warrington, PA).

The HIV-1 non-nucleoside reverse transcriptase inhibitors, UC781 and TMC120 were obtained by custom synthesis from Albany Molecular Research, Inc., Albany, NY. Zinc finger inhibitors #89 and #247 were a gift from Dr. Ettore Appella and Dr. Marco Schito (National Cancer Institute, Bethesda, MD). Stabilite SD60 Polyglycitol, hydroxypropyl methylcellulose E4M and Avicel PH 105, respectively, were from SPI Polyols, New Castle, DE, Dow Chemical Co., Midland, MI and the FMC Biopolymer Corporation, Philadelphia, PA. SP was purchased from Vital Products, Inc., Boynton Beach, FL. HIV-1 IIIB and BaL were from Advanced Biotechnologies, Inc., Colombia, MD. HeLa-CD4-LTR-β-gal, MAGI-CCR5 and TZM-bl cells were obtained from the AIDS Reagent and Reference Reagent Program (operated by McKesson BioServices, Rockville, MD) and contributed by Drs. M. Emerman, J. Overbaugh and J. C. Kappes and X. Wu (Tranzyme, Inc.), respectively. Dulbecco's modified Eagle medium (DMEM) was from GIBCO Invitrogen Corporation, Carlsbad, CA. The Galacto-Light Plus chemiluminescence reporter assay for β-galactosidase was from Applied Biosystems, Foster City, CA.

### Inhibition of infection by anti-HIV-1 compounds in the presence or absence of seminal plasma

Seventy μl of serially two-fold diluted compounds in DMEM medium (final concentrations after dilution: 1.25 to 10,000 μg/ml) were mixed with 70 μl of HIV-1 IIIB and BaL, respectively, and 70 μl of either SP or DMEM medium. The mixtures were added to TZM-bl indicator cells (in 96-well plates) which can be infected by both X4 and R5 HIV-1, enabling quantitative analysis of HIV-1 infection using either β-galactosidase (β-gal) or luciferase as a reporter [19]. After 90 min at 37°C, the virus (and SP) containing supernatants were removed from the cells. The cells were washed once with DMEM medium, supplemented with the same medium, and incubated for 48 hr at 37°C. The removal of SP after 90 min was necessary to avoid problems caused by the cytotoxicity of SP evident after prolonged incubation [20-22]. Finally, the culture supernatants were removed and the cells were washed once with phosphate buffered saline, pH 7.2 (PBS). Subsequently, 50 μl of lysis buffer from the Galacto-Light Plus kit were added to the wells for 1 hr at 20°C. Aliquots (20 μl) of the cell lysates were transferred into wells of 96-well microplates and β-gal was quantitated using the Galacto-Light Plus System chemiluminescence reporter assay in a MicroLight ML 2250 luminometer (Dynatech Laboratories, Inc. Chantilly, VA). The concentration of viruses was selected so as to provide a readout of ≤ 70 in the absence of SP and drug, respectively. The percentage of inhibition was calculated using the Microsoft Excel computer program. ED_50 _values were calculated using an online computer program [23]. The inhibitory activity of the compounds in the absence of SP was measured also at a lower (1/6) virus dose (readout ≤ 20) to determine whether or not this would result in higher ED_50 _values. This was not the case, confirming that the presence of SP (causing partial virus inactivation or inhibition) truly diminishes the inhibitory activity of the polymers under investigation.

### Measurements of HIV-1 infectivity

The following two formulations were tested for virucidal activity against HIV-1 IIIB and BaL, respectively, in the presence of SP: (1) Aquateric, 18%; glycerol, 58%; Stabilite SD60, 20%; Hydroxypropyl methylcellulose E4M 1%, Avicel PH 105, 3%; and (2) 18% Aquateric in a universal placebo gel [24].

Two-fold serial dilutions of HIV-1 IIIB treated with Aquateric formulations, and separated from these polymers by precipitation with 3% PEG or by centrifugation at 14,000 rpm for 1 h, and control virus (100 μl), respectively, were added to HeLa-CD4-LTR-β-gal cells which had been plated a day before infection in 96-well plates at 1 × 10^4 ^cells/well in 100 μl of DMEM medium containing 10% fetal bovine serum (FBS). After incubation at 37°C for 48 h, the culture supernatant fluids were removed and the cells washed once with PBS. β-gal in the cells was measured as described above. The infectivity of treated and control HIV-1 BaL was measured by the same method except that MAGI-CCR5 cells were used.

Measurement of HIV-1 infectivity in seminal fluid is impeded by the cytotoxicity of SP [20,21] contributed to by spermine [22]. Therefore, HIV-1 was separated from SP ingredients by precipitation with 3% PEG or centrifugation (14,000 rpm for 1 h) and the infectivity of the resuspended pellets was measured. Such cytotoxic effects were minimized by using for titrations of virus infectivity pellets after precipitation with 3% PEG or after centrifugation (14,000 rpm for 1 h).

### Micromethod for CAP quantitation

CAP in the form of a complex with ruthenium red was quantitated spectrophotometrically [25].

## Results

### The HIV-1 inhibitory activity of several polymeric candidate microbicides is diminished in the presence of seminal plasma

**Figure 1:**
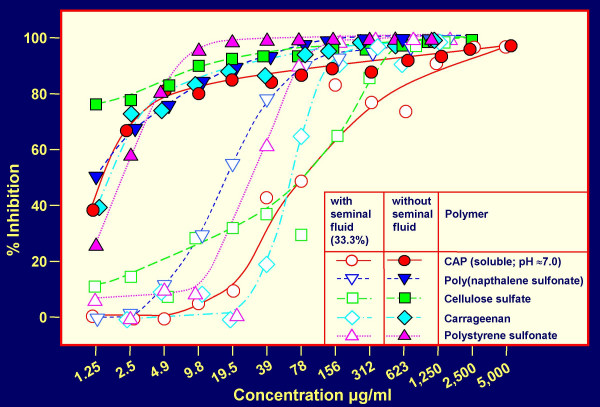
**Inhibition of HIV-1 IIIB infection by negatively charged polymers in the presence (33.3%; v/v), and absence of seminal plasma, respectively**. TZM-bl indicator cells and β-galactosidase readout were used for quantitative analysis of HIV-1 infection.

Inhibition of HIV-1 IIIB (a virus utilizing CXCR4 as cellular coreceptor = X4 virus) and HIV-1 BaL (a virus utilizing CCR 5 as cellular receptor = R5 virus) [8,26] infection, respectively, of TZM-bl cells [19] by polymeric candidate microbicides in the absence or presence of SP (final concentration 33.3%) was investigated. The polymers included: carrageenan, poly(naphthalene sulfonate), cellulose sulfate, cellulose acetate 1,2-benzene dicarboxylate (CAP) and polystyrene sulfonate. Dose response curves for inhibition of HIV-1 IIIB infection (Fig. 1) show the suppressive effect of SP on the virus inhibitory activities of the polymers. Similar results were obtained for HIV-1 BaL (data not shown). The results are summarized in Table 1 showing values for 50% inhibition of infection (ED_50_) corresponding to the distinct polymers. The ED_50 _values are consistently higher for HIV-1 BaL than for HIV-1 IIIB. This is consistent with lower positive charges on V3 loops of R5 gp120 envelope glycoproteins as compared with X4 gp120, leading to diminished binding of negatively charged polymers [27,28]. The presence of SP led to a ≈ 4 to ≈ 73-fold increase of ED_50_, resulting in ED_50 _values for HIV-1 BaL (a representative of more frequently sexually transmitted R5 viruses [8,26]) > 100 μg/ml. This raises the question whether or not sufficient concentrations of the respective inhibitors can be reached in the vaginal environment at which cells playing a major role in the virus replicative cycle (dendritic cells, macrophages and CD4+ T cells) occur [11].

**Table 1:** Decreased anti-HIV-1 activity of polymeric candidate microbicides in the presence of human seminal plasma

Polymer	HIV-1 IIIB			HIV-1 BaL		
	
	ED_50 _μg/ml	ED_50SP _μg/ml	ED_50SP_: ED_50 _ratio	ED_50 _μg/ml	ED_50SP _μg/ml	ED_50SP_: ED_50 _ratio
Poly (naphthalene sulfonate)	1.1	17.3	15.7	37.5	141	3.8
Cellulose sulfate	1.6	64.7	40.4	7.1	401	56.5
Carrageenan	1.4	63.4	45.6	6.3	149	23.7
Cellulose acetate 1,2-benzenedi-carboxylate	1.0	72.7	72.7	9.8	141	14.4
Polystyrene sulfonate	2.2	36.1	16.4	12.8	389	30.4

SP = seminal plasma added

In contrast with the polymeric candidate microbicides, the anti-HIV-1 activities of reverse transcriptase inhibitors UC781 and TMC120, and of zinc finger inhibitors #89 and #247, respectively, were not affected by the presence of SP (data not shown). This will support confidence for results of efficacy tests for these antiretroviral drugs in animal model systems for male to female virus transmission. The presence of SP alone (33.3 to 80%) resulted in 4- to 22-fold, and 2- to 10-fold inhibition of HIV-1 IIIB and BaL infection, respectively, in different experiments.

### Direct HIV-1 inactivation in the presence of seminal plasma

Anionic polymeric candidate microbicides are thought to interfere with HIV-1-cell interactions involving cellular receptors either directly in productive infection (CD4 and CXCR4/CCR5) or in cell to cell transfer of virus (DC-SIGN etc.) [11]. However, some polymers rapidly (≤ 5 min) inactivate HIV-1. Under these circumstances, there is no need for the antiviral compounds to reach cells involved in virus dissemination or infection, the virus being rendered non-infectious before encountering cellular/tissue surfaces. Among the aforementioned polymers, cellulose sulfate, polystyrene sulfonate, poly(naphthalene sulfonate) (at a concentration of 10 mg/ml; at concentrations ≤ 1 mg/ml the compounds may not be virucidal [29]), CAP (micronized), as well as the acidifying formulation, BufferGel, inactivate not only X4 viruses but also the R5 virus, HIV-1 BaL [30]. Poly(naphthalene sulfonate) and CAP, respectively, rapidly elicit in the virus envelope the formation of "dead-end" gp41 six-helix bundles rendering the virus irreversibly incapable to fuse with susceptible target cells, a prerequisite for infection [30]. CAP being the only candidate microbicide provided in a water insoluble micronized form, and therefore, very unlikely to penetrate into tissues or cells [31], was selected for further studies regarding the effect of SP on its virus inactivating properties.

**Figure 2:**
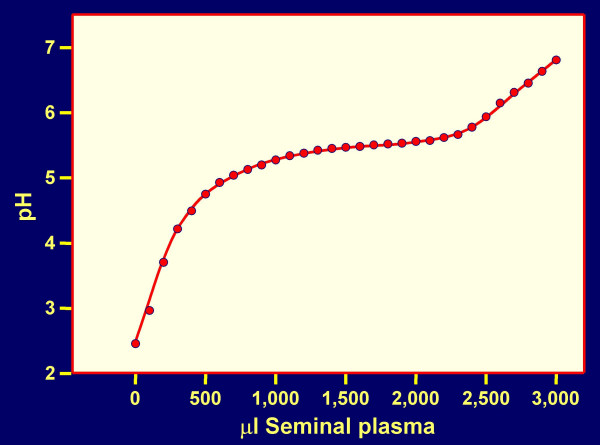
**pH changes caused by addition of seminal plasma to 180 mg Aquateric**. Increasing volumes of seminal plasma were added to 180 mg Aquateric, and pH was measured. Aquateric is a micronized form of CAP and consists of ≈ 67% CAP and ≈ 33% poloxamer + distilled acetylated monoglycerides. CAP formulations have been designed to contain 180 mg Aquateric per gram of formulation.

**Figure 3:**
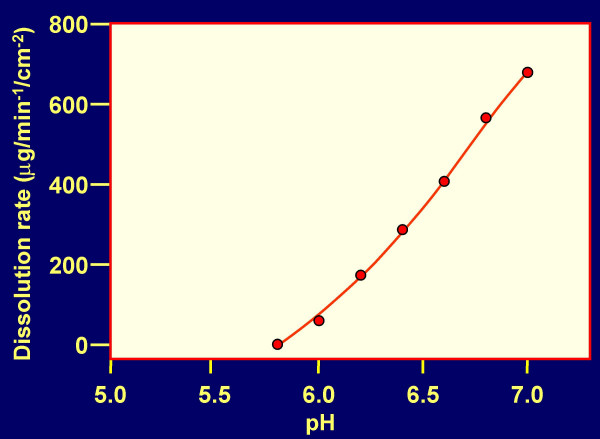
**Dissolution rate of CAP as a function of pH**. Data are derived from J. Spitael and R. Klinget: Solubility and dissolution rate of enteric polymers, *Acta Pharmaceutica technologica *1979; S7:163–168 [56].

**Figure 4:**
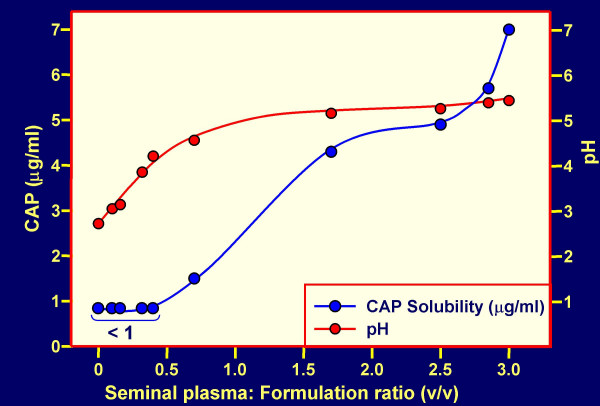
**CAP solubility in seminal plasma-Aquateric formulation mixtures**. Increasing volumes of seminal plasma were added to one ml of formulation 1 (see Methods section). The pH was measured; the mixtures were centrifuged to pellet most of Aquateric and CAP in the supernatant fluids was quantitated [25].

**Figure 5:**
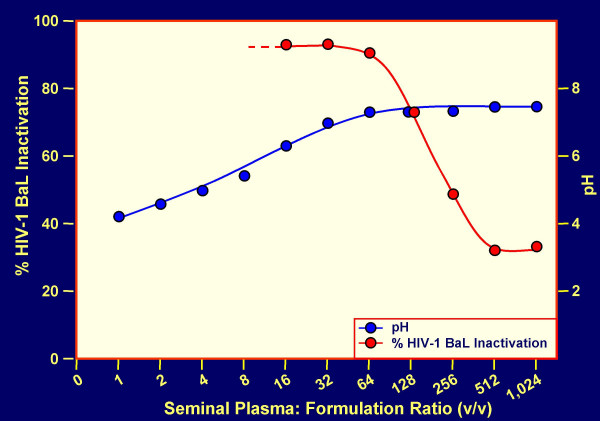
**HIV-1 BaL inactivation in seminal plasma-Aquateric formulation mixtures**. Increasing volumes of seminal plasma were added to aliquots of formulation 2 (see Methods section). After 5 min at 37°C, the samples were cooled in ice, and residual infectivity was measured. The decrease in extent of virus inactivation reflects both dilutions of the Aquateric formulation by seminal plasma and a concomitant pH increase. Samples diluted ≤ 8-fold were cytotoxic due to low pH, and the percentage of virus inactivation could not be determined.

CAP formulations (expected to contain about 240 to 480 mg CAP per dose for human use) have a good acidic buffering capacity, are non-toxic to vaginal and cervical explants or reconstituted tissues [32], and provide a pH ≤ 5.5 if the volume of SP is ≤ 1.8 ml per 100 mg CAP (Fig. 2). CAP remains water insoluble under these conditions (Fig. 3) and will remain in the form of particles about 1 micron in diameter. Changes in pH and CAP solubility during the course of addition of SP to formulated CAP (≈ 120 mg/ml) are shown in Fig. 4. It is evident that even at an excess of SP over the volume of the formulation used (3:1), the pH remains < 5.5 and the maximum concentration of CAP becoming soluble is ≤ 7 μg/ml. This concentration is sufficient for partial inhibition of infection by the laboratory strain HIV-1 IIIB [33] but insufficient for inhibition of most other HIV-1 strains [34]. Thus, the predominant contribution to anti-HIV-1 activity of formulated CAP is expected to be attributable to the insoluble micronized particles even at an excess of SP. These particles adsorb both X4 and R5 viruses, elicit 6-helix bundle formation in their envelope glycoproteins and render the viruses non-infectious [30]. Both HIV-1 IIIB and BaL are inactivated within 5 min at 37°C by CAP in micronized form even in the presence of a volume excess of SP (Table 2). Similar results were obtained with an Aquateric formulation in a "universal placebo" gel [24] (Fig. 5). Virucidal activity against HIV-1 BaL was still detectable at relatively high dilutions of the formulation in SP (resulting in pH ≥ 7.0), not expected to occur *in vivo*. The virucidal activity of CAP was similar to that observed in the absence of SP (see Fig. 2 in our earlier publication) [35]. In contrast, acidic pH alone is much less virucidal than CAP in micronized form, the half-life for virus infectivity being 6, 10 and >120 min at pH 3.5, 4.0 and 4.5, respectively [36] and fails to elicit "dead-end" six-helix bundle formation in HIV-1 gp41 (data not shown). Thus, combination of a low pH buffering system with a compound targeted to functionally important sites on HIV-1 envelope glycoproteins (e.g. CD4 and/or CXCR4/CCR5 coreceptor binding sites, and gp41 regions involved in 6-helix bundle formation) provides an attractive approach for development of virucidal microbicides. In the case of CAP, the low-pH buffering capacity is an inherent property of the polymer.

**Table 2:** Inactivation of HIV-1 IIIB and BaL by formulated Aquateric (18%) (5 min, 37°C) in the presence of seminal plasma

Seminal Plasma: Formula Ratio		% Virus Inactivation			
		
		HIV-1 IIIB		HIV-1 BaL	
Ratio	pH	Aquateric Pellet	Supernatant	Aquateric Pellet	Supernatant

0.33	3.9	≥ 99.8	≥ 99.8	≥ 99.6	≥ 99.6
1.0	4.8	≥ 99.8	≥ 99.8	≥ 99.6	≥ 99.6
3.0	5.43	≥ 99.8	≥ 99.8	≥ 99.6	≥ 99.6

Virus concentrates of HIV-1 IIIB and Bal, respectively, were diluted 200-fold in seminal plasma and then mixed with Aquateric formulation 1 (see Methods section) at volume ratios indicated in the Table. After incubation for 5 min at 37°C, the samples were cooled in ice and centrifuged at 10,000 rpm for 10 min. The supernatant fluids were neutralized by addition of 1 M Na_2_HPO_4_. The pellets containing Aquateric with adsorbed virus were resuspended and brought to pH 7.0 by addition of 1 M Na_2_HPO_4_. Both the supernatants and the redissolved pellets were mixed with a solution of PEG 8000 (final concentration 3%). The resulting pellets were resuspended in DMEM medium and tested for infectivity as described in the Methods Section. Virus preparations diluted in seminal fluid but not treated with the Aquateric formulation represented controls.

## Discussion

Results presented here indicate that SP caused a decrease in the HIV-1 inhibitory activity of all polymers being considered as candidate microbicides while inhibition of virus infection caused by reverse transcriptase and zinc finger inhibitors remained unaffected. Semen contains the polyamines spermine, spermidine and putrescine [37,38] which are positively charged at neutral pH while the polymeric anti-HIV-1 inhibitors all have negative charges. Thus, complex formation between the polyamines and the anti-HIV-1 polymers seems likely. This is supported by the observed complex formation between polyamines and sulfonic and carboxylic polyanions [39,40]. In agreement with this, SP interfered with the inhibitory effect of a selected candidate microbicide, cellulose sulfate, on binding of monoclonal antibodies to the positively charged V3 loop of HIV-1 IIIB gp120 [41] (data not shown). This interference was completely abrogated by addition of sodium maleate with a negative charge in the maleate moiety, forming complexes with polyamines. However, maleate failed to restore the inhibitory activity of the polymeric candidate microbicides against HIV-1 infection to levels observed in the absence of SP (data not shown). Thus, electrostatic interactions between SP polyamines and the polyanions tested seem to provide an incomplete explanation for the suppression of anti-HIV-1 activities of polymeric candidate microbicides by SP.

The decreased anti-HIV-1 inhibitory activity *in vitro *of polymeric candidate microbicides in the presence of SP suggest that these compounds may become less effective *in vivo *in animal models and human clinical trials than would be expected from macaque efficacy studies using a semen-free virus challenge [5,6]. This possibility confirms the need for development of combination microbicides in which candidate polymers would be supplemented with anti-HIV-1 compounds remaining fully effective in the presence of SP, and preferably having a mechanism of action distinct from that of the polymeric microbicides. The simplest approach towards this goal, which may also bypass potential regulatory hurdles resulting from two active ingredients in a single formulation, is to formulate the anti-HIV-1 polymers in buffer systems maintaining an acidic pH (similar to that of a normal vaginal environment) even in the presence of semen, and causing inactivation of HIV-1 and other sexually transmitted disease (STD) pathogens. Such buffering bioadhesive formulations include ACIDFORM [42,43] based on small molecule components, and preferably polymers with built-in acidic buffering capacity: Carbomer 974P (BufferGel) [44] and CAP (Fig. 2, 3, 4, 5; Table 1).

To fully appreciate the role of SP ingredients on sexual transmission of HIV-1, one should consider also the observations that they appear to cause a decrease of infectious virus titer (see Results; [36,45]). Major contributors to this phenomenon are probably products resulting from oxidation of SP polyamines by diamine oxidase [46] which is also present in this biological fluid [47,48]. The oxidation is augmented by peroxidase [46]. Peroxidase is present in a healthy vaginal environment [49,50]. This suggests that a combination of SP ingredients and a "normal" vaginal environment might provide a natural defense system against infection by HIV-1 and other STD pathogens and might contribute to the low incidence of HIV-1 transmission per unprotected coital act [8]. In fact, several viruses and parasites were shown to become inactivated by polyamine oxidation products [51-55]. Efforts to augment this natural defense mechanism might potentially become part of the microbicide development strategy.

## Conclusion

Candidate anti-HIV-1 topical microbicides have been evaluated for virus inhibitory activities *in vitro *and efficacy in animal models mostly without considering the possible effects semen/seminal plasma may have on the ultimate performance of these compositions. Studies in macaques challenged vaginally by SIV or SHIV, after application of anti-HIV-1 compounds, indicated that drug doses required for protection against infection exceeded by several orders of magnitude concentrations sufficient for blocking virus replication *in vitro*. Since semen/seminal plasma is a vehicle for male to female sexual transmission of the virus, it is of crucial interest to determine its potential impact on HIV-1 inhibitory activities of candidate products. Such studies are difficult, if not impossible, in animal models. Therefore, at least a relevant assessment in *in vitro *systems is called for. Results presented here indicate that the anti-HIV-1 activity of synthetic polymers, acting as HIV-1 entry inhibitors (carrageenan, cellulose sulfate, poly(naphthalene sulfonate), polystyrene sulfonate and CAP in water soluble form) is greatly diminished in the presence of SP. The impact of this finding on performance of these candidate microbicides *in vivo *remains unknown. On the other hand, HIV-1 inhibition by reverse transcriptase inhibitors, UC781 and TMC120 and by zinc finger inhibitors #89 and #247, respectively, was not affected by SP. Combination of polymeric HIV-1 entry inhibitors with active compounds having a distinct mechanism of action, and reliance on polymers having direct virucidal activity and built-in low pH buffering capacity (contributing to virus inactivation) (CAP in micronized form; BufferGel) are expected to overcome potential problems resulting from interference of semen components with the performance of some microbicides being considered for, or already in clinical trials.

## Competing interests

The author(s) declare that they have no competing interests.

## Authors' contributions

Author 1 ARN developed the concepts representing the basis of the manuscript and designed most experiments; author 2 NS carried out most experiments and contributed to the development of experimental techniques; author 3 YYL did all the tissue culture work and infectivity assays. All authors have read and approved the final manuscript.

## Pre-publication history

The pre-publication history for this paper can be accessed here:



## References

[B1] UNAIDS (2006). 2006 Report on the global AIDS epidemic.

[B2] Miller CJ, Alexander NJ, Sutjipto S, Lackner AA, Gettie A, Hendrickx AG, Lowenstine LJ, Jennings M, Marx PA (1989). Genital mucosal transmission of simian immunodeficiency virus: animal model for heterosexual transmission of human immunodeficiency virus. J Virol.

[B3] Boadi T, Schneider E, Chung S, Tsai L, Gettie A, Ratterree M, Blanchard J, Neurath AR, Cheng-Mayer C (2005). Cellulose acetate 1,2-benzenedicarboxylate protects against challenge with pathogenic X4 and R5 simian-human immunodeficiency viruses. AIDS.

[B4] Otten RA, Adams DR, Kim CN, Jackson E, Pullium JK, Lee K, Grohskopf LA, Monsour M, Butera S, Folks TM (2005). Multiple vaginal exposures to low doses of R5 simian-human immunodeficiency virus: Strategy to study HIV preclinical interventions in nonhuman primates. J Infect Dis.

[B5] Weber J, Nunn A, O'Connor T, Jeffries D, Kitchen V, McCormack S, Stott J, Almond N, Stone A, Darbyshire J (2001). 'Chemical condoms' for the prevention of HIV infection: evaluation of novel agents against SHIV89.6PD in vitro and in vivo. AIDS.

[B6] Lewis MG, Wagner W, Yalley-Ogunro J, Greenhouse J, Burrier J, Profy AT (2001). Efficacy of PRO 2000 gel in a macaque model for vaginal HIV transmission: 1 AD/2/4=02/08/2001..

[B7] Jiang YH, Emau P, Cairns JS, Flanary L, Morton WR, McCarthy TD, Tsai CC (2005). SPL7013 gel as a topical microbicide for prevention of vaginal transmission of SHIV89.6P in macaques. AIDS Res Hum Retroviruses.

[B8] Shattock RJ, Moore JP (2003). Inhibiting sexual transmission of HIV-1 infection. Nat Rev Microbiol.

[B9] Veazey RS, Klasse PJ, Schader SM, Hu Q, Ketas TJ, Lu M, Marx PA, Dufour J, Colonno RJ, Shattock RJ, Springer MS, Moore JP (2005). Protection of macaques from vaginal SHIV challenge by vaginally delivered inhibitors of virus-cell fusion. Nature.

[B10] Lacey CJ, Wright A, Weber JN, Profy AT (2006). Direct measurement of in-vivo vaginal microbicide levels of PRO 2000 achieved in a human safety study. AIDS.

[B11] Stone A (2002). Microbicides: A new approach to preventing HIV and other sexually transmitted infections. Nat Rev Drug Discov.

[B12] Dezzutti CS, James VN, Ramos A, Sullivan ST, Siddig A, Bush TJ, Grohskopf LA, Paxton L, Subbarao S, Hart CE (2004). In vitro comparison of topical microbicides for prevention of human immunodeficiency virus type 1 transmission. Antimicrob Agents Chemother.

[B13] Borkow G, Parniak MA (2001). Anti-HIV-1 microbicide potential of the tight-binding nonnucleoside reverse trancriptase inhibitor UC781. AIDScience.

[B14] Di Fabio S, Van Roey J, Giannini G, van den Mooter G, Spada M, Binelli A, Pirillo MF, Germinario E, Belardelli F, de Bethune MP, Vella S (2003). Inhibition of vaginal transmission of HIV-1 in hu-SCID mice by the non-nucleoside reverse transcriptase inhibitor TMC120 in a gel formulation. AIDS.

[B15] International Partnership for Microbicides I (2005). A safety and tolerability study of dapvirine (TMC120) vaginal microbicide gel.

[B16] Turpin JA (2003). The next generation of HIV/AIDS drugs: novel and developmental antiHIV drugs and targets. Exp Rev Anti-infective Therapy.

[B17] Schito ML, Goel A, Song Y, Inman JK, Fattah RJ, Rice WG, Turpin JA, Sher A, Appella E (2003). In vivo antiviral activity of novel human immunodeficiency virus type 1 nucleocapsid p7 zinc finger inhibitors in a transgenic murine model. AIDS Res Hum Retroviruses.

[B18] Goel A, Mazur SJ, Fattah RJ, Hartman TL, Turpin JA, Riche WG, Appella E, Inman JK (2002). Benzamide-based thiolcarbamates: A new class of HIV-1 NCp7s inhibitors. Bioorg Med Chem.

[B19] Wei X, Decker JM, Liu H, Zhang Z, Arani RB, Kilby JM, Saag MS, Wu X, Shaw GM, Kappes JC (2002). Emergence of resistant human immunodeficiency virus type 1 in patients receiving fusion inhibitor (T-20) monotherapy. Antimicrob Agents Chemother.

[B20] Rasheed S, Li Z, Xu D (1995). Human immunodeficiency virus load. Quantitative assessment in semen from seropositive individuals and in spiked seminal plasma. J Reprod Med.

[B21] Krieger JN, Coombs RW, Collier AC, Ho DD, Ross SO, Zeh JE, Corey L (1995). Intermittent shedding of human immunodeficiency virus in semen: implications for sexual transmission. J Urol.

[B22] Allen RD, Roberts TK (1987). Role of spermine in the cytotoxic effects of seminal plasma. Am J Reprod Immunol Microbiol.

[B23] Lattmann M (1999). Nichtlineare Interpolation (Non-linear Interpolation).

[B24] Tien D, Schnaare RL, Kang F, Cohl G, McCormick TJ, Moench TR, Doncel G, Watson K, Buckheit RWJ, Lewis MG, Schwartz J, Douville K, Romano JW (2005). In vitro and in vivo characterization of a potential universal placebo designed for use in vaginal microbicide clinical trials. AIDS Res Hum Retroviruses.

[B25] Neurath AR, Strick N (2001). Quantitation of cellulose acetate phthalate in biological fluids as a complex with ruthenium red. Anal Biochem.

[B26] Shattock RJ, Doms RW (2002). AIDS models: Microbicides could learn from vaccines. Nat Med.

[B27] Moulard M, Lortat-Jacob H, Mondor I, Roca G, Wyatt R, Sodroski J, Zhao L, Olson W, Kwong PD, Sattentau QJ (2000). Selective interactions of polyanions with basic surfaces on human immunodeficiency virus type 1 gp120. J Virol.

[B28] Jiang S (1997). HIV-1--co-receptors binding. Nat Med.

[B29] Fletcher PS, Wallace GS, Mesquita PMM, Shattock RJ (2006). Candidate polyanion microbicides inhibit HIV-1 infection and dissemination pathways in human cervical explants. Retrovirology.

[B30] Neurath AR, Strick N, Li YY (2002). Anti-HIV-1 activity of anionic polymers: A comparative study of candidate microbicides. BMC Infect Dis.

[B31] Olmsted SS, Padgett JL, Yudin AI, Whaley KJ, Moench TR, Cone RA (2001). Diffusion of macromolecules and virus-like particles in human cervical mucus. Biophys J.

[B32] Trifonova RPJM, Fichorova RN (2006). Biocompatibility of solid dosage forms of anti-HIV-1 microbicides with the human cervico-vaginal mucosa modeled ex vivo. Antimicrobial Agents and Chemotherapy.

[B33] Neurath AR, Strick N, Li YY, Lin K, Jiang S (1999). Design of a "microbicide" for prevention of sexually transmitted diseases using "inactive" pharmaceutical excipients. Biologicals.

[B34] Lu H, Zhao Q, Wallace G, Liu S, He Y, Shattock R, Neurath AR, Jiang S (2006). Cellulose acetate 1,2-benzenedicarboxylate inhibits infection by cell-free and cell-associated primary HIV-1 isolates. AIDS Res Hum Retroviruses.

[B35] Neurath AR, Strick N, Li YY, Debnath AK (2001). Cellulose acetate phthalate, a common pharmaceutical excipient, inactivates HIV-1 and blocks the coreceptor binding site on the virus envelope glycoprotein gp120. BMC Infect Dis.

[B36] O'Connor TJ, Kinchington D, Kangro HO, Jeffries DJ (1995). The activity of candidate virucidal agents, low pH and genital secretions against HIV-1 in vitro. Int J STD AIDS.

[B37] Janne J, Holtta E, Haaranen P, Elfving K (1973). Polyamines and polyamine-metabolizing enzyme activities in human semen. Clinica Chimica Acta.

[B38] Terazawa K, Taktori T (1983). Enzymic fluorometry of spermine in seminal fluid. Jpn J Exp Med.

[B39] Crea F, De Robertis A, De Stefano C, Sammartano S, Gianguzza A, Piazzese D (2001). Binding of acrylic and sulphonic polyanions by open-chain polyammonium cations. Talanta.

[B40] De Robertis A, De Stefano C, Gianguzza A, Sammartano S (1999). Binding of polyanions by biogenic amines. III. Formation and stability of protonated spermidine and spermine complexes with carboxylic ligands. Talanta.

[B41] Skinner MA, Ting R, Langlois AJ, Weinhold KJ, Lyerly HK, Javaherian K, Matthews TJ (1988). Characteristics of a neutralizing monoclonal antibody to the HIV envelope glycoprotein. AIDS Res Hum Retroviruses.

[B42] Garg S, Anderson RA, Chany CJ, Waller DP, Diao XH, Vermani K, Zaneveld LJ (2001). Properties of a new acid-buffering bioadhesive vaginal formulation (ACIDFORM). Contraception.

[B43] Amaral E, Perdigao A, Souza MH, Mauck C, Waller DZL, Faundes A (2006). Vaginal safety after use of a bioadhesive, acid-buffering, microbicidal contraceptive gel (ACIDFORM) and a 2% nonoxynol-9 product. Contraception.

[B44] Olmsted SS, Khanna KV, Ng EM, Whitten ST, Johnson ONIII, Markham RB, Cone RA, Moench TR (2005). Low pH immobilizes and kills human leukocytes and prevents transmission of cell-associated HIV in a mouse model. BMC Infect Dis.

[B45] Shugars DC (1999). Endogenous mucosal antiviral factors of the oral cavity. J Inf Dis.

[B46] Klebanoff SJ, Kazazi F (1995). Inactivation of human immunodeficiency virus type 1 by the amine oxidase-peroxidase system. J Clin Microbiol.

[B47] Holtta E, Pulkkinen P, Elfving K, Janne J (1975). Oxidation of polymines by diamine oxidase from seminal plasma. Biochem J.

[B48] Le Calve M, Segalen J, Quernee D, Lavault MT, Lescoat D (1995). Diamine oxidase activity and biochemical markers in human seminal plasma. Hum Reprod.

[B49] Klebanoff S, Hillier SL, Eschenbach DA, Waltersdorph AM (1991). Control of the microbial flora of the vagina by H2O2-generating lactobacilli. J Inf Dis.

[B50] Boris S, Barbes C (2000). Role played by lactobacilli in controlling the population of vaginal pathogens. Microbes Infect.

[B51] Katz E, Goldblum T, Bachrach U, Goldblum N (1967). Antiviral action of oxidized spermine. Inactivation of certain animal virus. Isr J Med Sci.

[B52] Bachrach U, Don S (1970). Inactivation of influenza and Newcastle disease viruses by oxidized spermine. Isr J Med Sci.

[B53] Bachrach U, Don S (1971). Inactivation of myxoviruses by oxidized polyamines. J Gen Virol.

[B54] Bachrach U, Rosenkovitch E (1972). Effect of oxidized spermine and other aldehydes on the infectivity of vaccinia virus. Applied Microbiology.

[B55] Morgan DML, Bachrach U, Assaraf YG, Harari E, Golenser J (1986). The effect of purified aminoaldehydes produced by polyamine oxidation on the development in vitro of Plasmodium falciparum in normal and glucose-6-phosphate-dehydrogenase-deficient erythrocytes. Biochem J.

[B56] Spitael J, Kinget R (1979). Solubility and dissolution rate of enteric polymers. Acta Pharmaceutica Technologica.

